# Instance segmentation for the fine detection of crop and weed plants by precision agricultural robots

**DOI:** 10.1002/aps3.11373

**Published:** 2020-07-28

**Authors:** Julien Champ, Adan Mora‐Fallas, Hervé Goëau, Erick Mata‐Montero, Pierre Bonnet, Alexis Joly

**Affiliations:** ^1^ Institut national de recherche en informatique et en automatique (INRIA) Sophia‐Antipolis, ZENITH team Laboratory of Informatics Robotics and Microelectronics–Joint Research Unit 34095 Montpellier CEDEX 5 France; ^2^ School of Computing Costa Rica Institute of Technology Cartago Costa Rica; ^3^ AMAP University of Montpellier CIRAD CNRS INRAE IRD Montpellier France; ^4^ CIRAD UMR AMAP Montpellier France

**Keywords:** autonomous robot, convolutional neural network, deep learning, digital agriculture, plant detection, weed electrification

## Abstract

**Premise:**

Weed removal in agriculture is typically achieved using herbicides. The use of autonomous robots to reduce weeds is a promising alternative solution, although their implementation requires the precise detection and identification of crops and weeds to allow an efficient action.

**Methods:**

We trained and evaluated an instance segmentation convolutional neural network aimed at segmenting and identifying each plant specimen visible in images produced by agricultural robots. The resulting data set comprised field images on which the outlines of 2489 specimens from two crop species and four weed species were manually drawn. We adjusted the hyperparameters of a mask region‐based convolutional neural network (R‐CNN) to this specific task and evaluated the resulting trained model.

**Results:**

The probability of detection using the model was quite good but varied significantly depending on the species and size of the plants. In practice, between 10% and 60% of weeds could be removed without too high of a risk of confusion with crop plants. Furthermore, we show that the segmentation of each plant enabled the determination of precise action points such as the barycenter of the plant surface.

**Discussion:**

Instance segmentation opens many possibilities for optimized weed removal actions. Weed electrification, for instance, could benefit from the targeted adjustment of the voltage, frequency, and location of the electrode to the plant. The results of this work will enable the evaluation of this type of weeding approach in the coming months.

World population growth in recent decades has required the increased and intensified use of agricultural lands to enhance global production and productivity (Tilman et al., 2011). This has resulted in the massive use of phytosanitary products, which are used to control crop diseases and pests, fight deficiencies through soil enrichment, and manage weeds (Matson et al., 1997). Weeds are often recognized as the most serious threat to organic agricultural production (Bàrberi, 2002), and their management can have a huge economic cost at the national level; for example, Pimentel et al. (2000) estimated that weeds decrease crop yields by 12% in the United States, which represents approximately US$32 billion in lost crop production annually. The implementation of efficient weed management methods is therefore essential for maintaining economically balanced agricultural activities. To better preserve our environment and contribute to the development of sustainable agriculture, we need to rethink some agricultural practices and change current paradigms. The reduction of phytosanitary products is necessary to fight soil depletion and biodiversity loss, and to better meet societal expectations and the new regulations under development (such as the Eco‐Phyto Plan in France [https://agriculture.gouv.fr/encouraging‐results‐ecophyto‐plan‐reduction‐pesticide‐use (accessed 2 June 2020)]). This will require the replacement of current weed control solutions with more environmentally friendly solutions.

Several experimental solutions for robotization in the agricultural world avoid the extensive use of non‐selective phytosanitary products, in particular reducing herbicides with more targeted approaches (Bakker et al., 2006; Steward et al., 2019). These alternative approaches mainly use mechanical and thermal methods (Bond et al., 2003; Slaughter et al., 2008), although a few experimental approaches use electricity to kill weeds, such as those proposed by Diprose and Benson (1984), Vigneault et al. (1990), and Vigneault and Benoît (2001). This type of solution can have two advantages: the electricity does not affect the soil deeply, which preserves the integrity of the crop roots, and this technique uses less polluting energy than mechanical and/or thermal robot approaches.

Regardless of which weeding solution is considered, the accurate detection and identification of weed specimens is a major challenge to optimizing yield. The electrification approach in particular requires great precision; the use of a high‐voltage electrical head for weed control requires the positioning of the head in the immediate proximity, or in contact with a weed for effective application (Nolte et al., 2018). This electrical head must be only a few centimeters from the target plant and closer to it than to any surrounding plant to allow the appropriate use of an electric arc. This arc passes through the target plant from the top of its stem to the end of one of its roots. The precise location of its stem is therefore essential to ensure efficiency.

The state‐of‐the‐art approach for locating crops and/or weeds in precision agriculture images (Milioto et al., 2018) is the use of *semantic segmentation* algorithms, i.e., algorithms that classify each pixel of the image as belonging either to the crop class or to the weed class. Most advanced algorithms make use of convolutional neural networks (CNNs) to achieve this task (Potena et al., 2016; Mortensen et al., 2017; Sa et al., 2017; Lottes et al., 2018). As discussed by Milioto et al. (2018), the advantage of semantic segmentation is that it provides a good trade‐off between accuracy and the speed of detection; however, this approach does not allow the detection of each specimen separately, nor does it allow the species identification of each specimen.

In this article, we study more advanced deep learning architectures that allow each specimen to be detected separately and classified among a potentially large number of crop or weed species. A first option would be to use an *object detection* neural network, such as the Fast R‐CNN architecture (Girshick, 2015), which has been shown to perform very well on a wide variety of tasks while remaining rather fast. Its output is in the form of bounding boxes and associated class labels surrounding each detected specimen; however, bounding boxes are not yet sufficiently accurate for some innovative weed removal processes such as electrification. The center of the bounding box may be used to place the electrode, but it may not necessarily correspond to a good action point. For an efficient electrification, an appropriate positioning would be the apical bud of the plant, which would be better approximated by the barycenter (i.e., the center of gravity) of the plant itself (see Fig. 1). This issue is especially important during the the plant’s earliest growth stages, when there is often a significant disproportion between the sizes of the very first and subsequent leaves. This disproportion is visible until the plant reaches a more advanced stage of development, in which all its new leaves are of equivalent size. More generally, the precise detection of the shape of the specimens rather than a bounding box could make it possible to target very specific action points.

**Figure 1:**
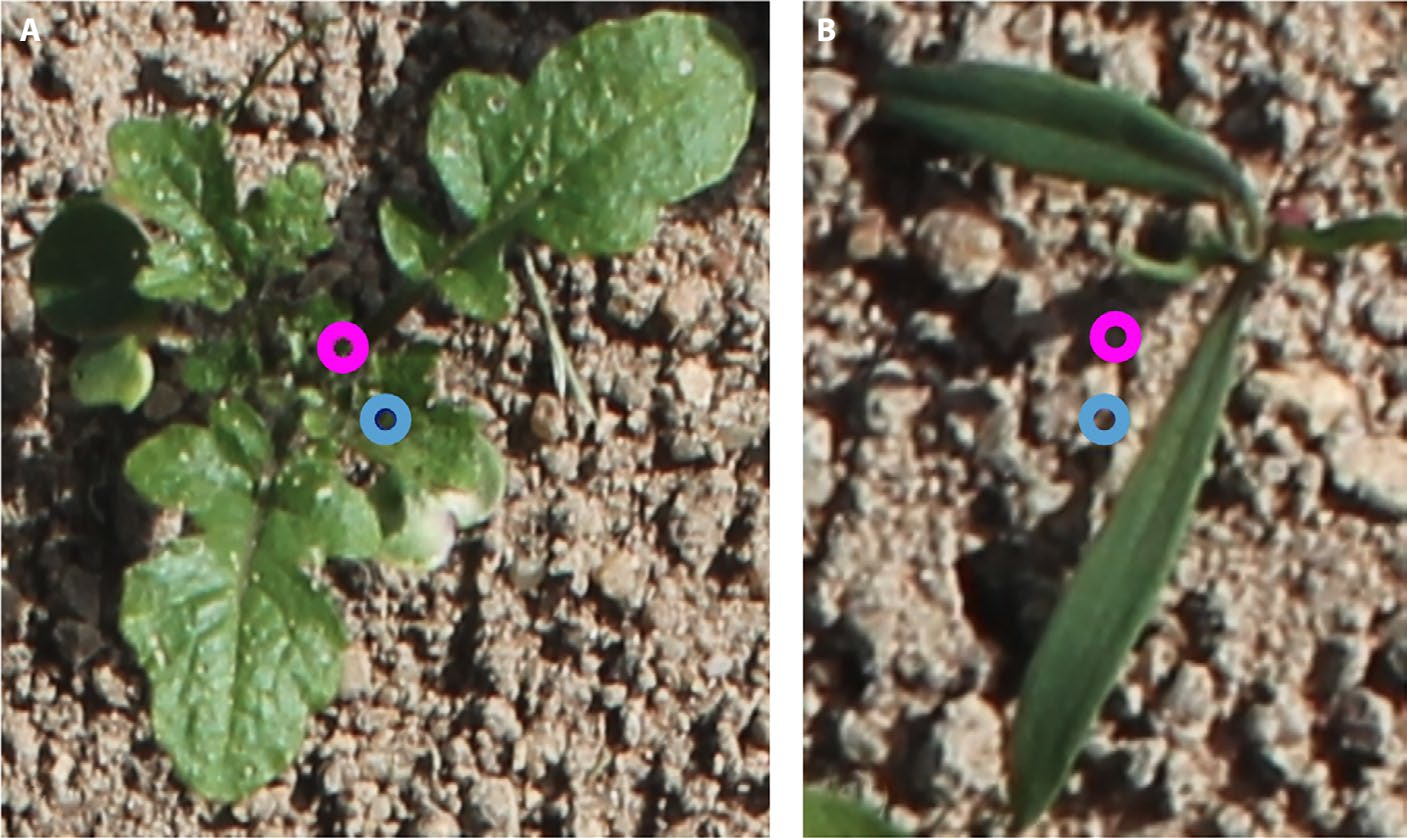
These images illustrate the importance of detecting the barycenter of the visible plant (pink circles) rather than the center of a bounding box around the plant (blue circles). The barycenter is usually much closer than the bounding box to the apical bud of the plant. (A) *Brassica nigra*. (B) A weed plant.

In this paper, we explore a new method for the accurate segmentation and identification of individual plants based on the use of instance segmentation CNNs. The output of such a network is in the form of *binary masks*, which encode the shape of each detected specimen and a class label associated with each mask (see Fig. 2 for an example). We used a Mask R‐CNN (He et al., 2017) architecture, for which we readjusted the hyperparameters to make them more adapted to the type of images and shapes encountered in precision farming. The model was then trained on a data set of 83 field images containing 2489 instances of crop and weed specimens that were manually segmented and identified by experts.

**Figure 2:**
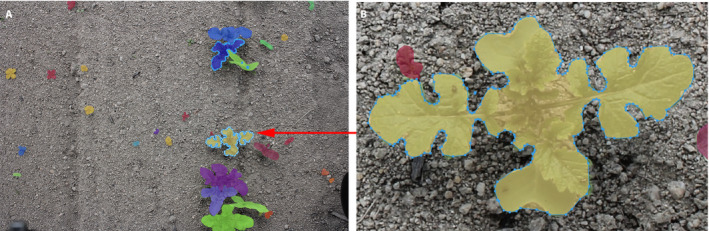
Example of a binary mask output from the instance segmentation convolutional neural network. (A) Illustration of the individual masks manually produced for one image of the training set. Each mask is annotated with the species name of the plant in the database management system. (B) Magnification of one individual mask of a *Brassica nigra* plant, showing all of the points (in blue) used to produce the outline of the mask.

The resulting model was tested on a prototype electric weeding robot in the field under semi‐controlled conditions. The experiments reported in this article concern two crop species that are representative of significantly different agricultural contexts in which the use of robots is developing. The first one, maize (*Zea mays* L.), is representative of a wide‐spaced field crop species with a tall, narrow shape. The second, the common bean (*Phaseolus vulgaris* L.), is representative of a shorter‐spaced vegetable crop species with a spreading shape. This work aims to answer the following questions: (i) Do the most advanced deep learning techniques allow for highly innovative weed control approaches such as the targeted electrification of weeds by autonomous robots? (ii) What spatial accuracy can be expected with respect to a particular task such as detecting the barycenter of each individual plant? (iii) Do the shapes and sizes of weeds affect the performance of this approach?

## METHODS

### Experimental site and species selection

This work was carried out at the Montoldre experimental site in central France (46°20′07″N, 3°26′50″E). This site, managed by the Institut national de recherche en sciences et technologies pour l’environnement et l’agriculture (IRSTEA), is located in a vast agricultural basin that offers many advantages for the experimentation of robotic solutions in agriculture such as: (i) large non‐rocky flat agricultural surfaces, (ii) substantial annual rainfall requiring only a small amount of irrigation, (iii) low exposure to intense meteorological events such as storms or hurricanes, (iv) a continental climate with smaller variations in temperature and humidity than other French regions (particularly in the south). Aerial and ground‐level views of this site are shown in Appendix S1.

**Figure 3:**
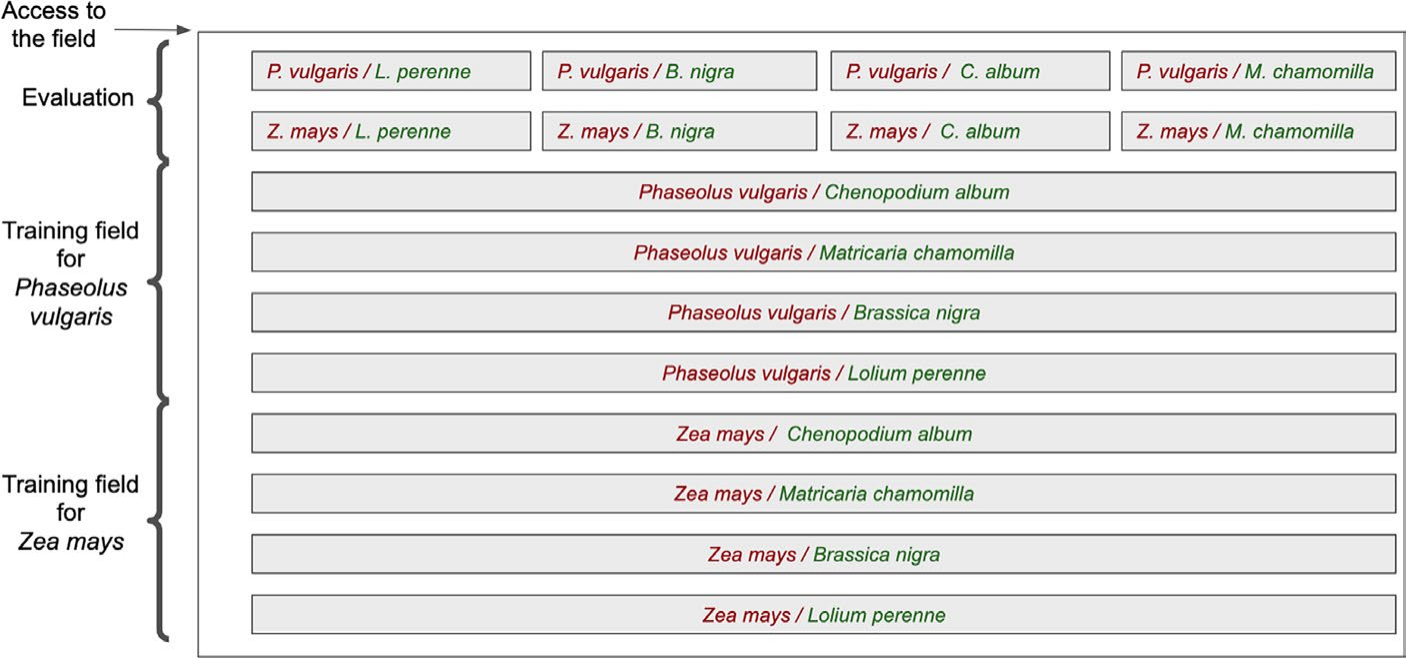
Map of the experimental field, showing the different combinations of crops and weed species.

In order to test our approach on different combinations of crops and weeds and their associated differences in morphological characteristics and management constraints, two crop species and four weed species were selected and seeded in combination, as shown in the plan on Fig. 3 (see also example plot in Appendix S1). The two crops used were maize, a large crop with wide plant spacings, and the common bean, a garden crop with a spreading shape and narrower spacings. We used two weed species with spreading shapes: the model species *Brassica nigra* (L.) W. D. J. Koch and the natural weed *Matricaria chamomilla* L. We also used two species with elongated shapes: the model species *Lolium perenne* L. and the natural weed *Chenopodium album* L.

These plants were grown following the experimental protocol presented in Appendix 1. The cultivation protocol was conducted from February 2019 to May 2019. Ground treatments (i.e., working the soil) with an automated spade were conducted from February to April, followed by a heat treatment designed to kill ungerminated seeds using localized high temperatures at the end of April. Sowings were conducted from the end of April to the middle of May, followed by the installation of a plastic film to protect the seedlings against birds and/or insects. Visual data were acquired in May 2019.

### Autonomous electrifier robot

Our experiment is based on the use of an agricultural robot, recently developed by ecoRobotix (https://ecorobotix.com). This robot, equipped with two large solar panels, is fully autonomous. In its initial configuration, the robot works without being controlled by a human operator, as its progression through the crop field is based on the use of its camera and real‐time kinematic global positioning system (RTK GPS). Two robotic arms apply a microdose of herbicide on detected weeds, based on a non‐crop detector that was centered on inter‐crop rows only. The machine can be fully controlled with a smartphone app. The technical information for this robot is provided in Table 1. In the context of the WeedElec (http://challenge‐rose.fr/en/projet/weedelec2017‐2/) initiative, the two sprayers on the robotic arms were removed and replaced by a high‐voltage electrical head. Two complementary cameras (Canon EOS 60D [Canon, Tokyo, Japan] equipped with a Canon EFS 24‐mm lens [Macro 0.16 m/0.52 ft], and a Canon EOS 1300D equipped with a Canon EF 40‐mm lens [Macro 0.3 m/0.98 ft]) were installed in the front of the robot below the solar panels. As illustrated in Appendix S2, the cameras were mounted 87 cm from the ground, just in front of the right robotic arm, in order to detect and identify weeds to be electrified.

**Table 1:** ecoRobotix robot specifications.

Characteristics	Details
Dimensions	2.20 m × 1.70 m × 1.30 m (width × length × height, camera folded down)
Weight	130 kg
Width of area covered	2 m
Speed	0.4 m/s (mean)
Space between crops	35–70 cm (adjustable)
Maximum height of crop	25 cm
Robotic arms	Executing 4000 movements per hour
Precision	<2 cm
Energy	Two photovoltaic panels, with 380‐W solar cells
Initial sensors	Megapixel camera, real‐time kinematic global positioning system (RTK GPS), compass
Communication	Short (WiFi) or long distance (mobile phone networks)
Soil humidity/wind requirements	Soil must not be too wet or viscous. Maximum wind 60 km/h at ground level

### Data set

To realistically evaluate our approach, the training samples and test samples that compose our data set were collected from separate crop rows of the experimental site (see Fig. 3). Eight plots (illustrated in Fig. 3, Appendix S1D) were reserved for the acquisition of training data, with each containing specimens of a single crop and a particular weed species. Naturally occurring weeds were not manually controlled, but prior heat treatment drastically limited their development. Each plot was composed of two parallel rows of crop (Appendix S1D). Two plots (at the top of Fig. 3), each divided into four sections containing a particular combination of crop and weed species, were reserved for the acquisition of test data. The training and test samples were acquired on two different days with a marked difference in the ambient brightness levels. The robot followed a linear and continuous path above a plot, and the images were acquired every 45 cm. The receptive field of each image is approximately equal to 80 cm width × 52 cm length.

A set of 2956 images was obtained from the training plots, from which a subset of 83 images were manually selected and fully annotated using a customized version of COCO Annotator (https://github.com/jsbroks/coco‐annotator), an online web tool aimed at recording manually drawn masks and related labels. For the test set, the same tool was used on a random selection of 21 images acquired from the test plots. Masks were produced at the individual plant level and annotated with one of the six targeted species, plus an additional category containing five additional weed species that grew naturally in the field and appeared in the images. It is important to emphasize that all visible crop and weed specimens were annotated in each image. Overall, this resulted in a data set of 2489 annotated plant specimens, each associated with a species name and a unique segmentation mask. Using this methodology, we obtained an average of 23.9 specimens per image. Details of the composition of the resulting training and test sets are provided in Table 2 (in particular, the number of annotated masks per crop and weed species).

**Table 2:** Number of object instances per species in the training and the test data sets.

Species name	Species type	Training data set	Test data set
*Zea mays* L.	Crops	98	28
*Phaseolus vulgaris* L.	Crops	405	49
*Brassica nigra* (L.) W. D. J. Koch	Cultivated weed	238	26
*Matricaria chamomilla* L.	Cultivated weed	362	333
*Lolium perenne* L.	Cultivated weed	290	46
*Chenopodium album* L.	Cultivated weed	228	34
Other weeds	Natural weed	868	502

### Deep learning detection and identification model

As discussed in the introduction, our fine‐grained detection method is based on the Mask R‐CNN architecture (He et al., 2017), which was selected for its robustness and demonstrated efficiency in instance segmentation tasks and challenges such as MS COCO (Microsoft Common Objects in Context; Lin et al., 2014). We used the Facebook Mask R‐CNN benchmark (Massa and Girshick, 2018) implemented with PyTorch (Paszke et al., 2017). This implementation offers different configurations for the backbone CNN and for instance segmentation. We chose ResNet‐50 (He et al., 2016) as the backbone CNN and Feature Pyramid Networks (Lin et al., 2017) for instance segmentation.

**Figure 4:**
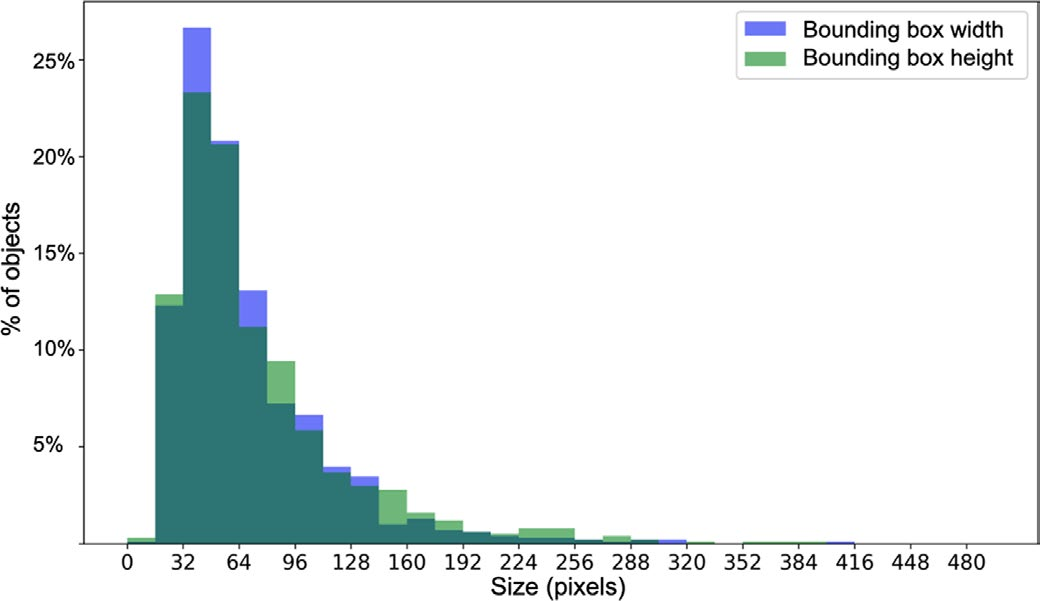
Statistics of the size of the bounding boxes for the plant instances in the training set.

To adjust the hyperparameters of this architecture, we calculated some statistics on the size of the masks in the training set (see Fig. 4). Based on this and hardware constraints, we used the following hyperparameter values: (i) Input image size: Images were resized so that their shorter edge was 1200 pixels and the longest one 2048 pixels. This allowed the model to be run in a reasonable time (about 4 h) on a standard graphics processing unit (with 8 Gb memory). (ii) Anchor size and stride: Anchors are the raw regions of interest used by the *region proposal network* to select the candidate bounding boxes for object detection. We chose their size to guarantee that 99% of the targeted objects were sized within the range between the minimal and the maximal anchor sizes. The anchor size values were therefore set to [32;64;128;256;512], the anchor stride values to [4;8;16;32;64], and the anchor ratios to [0.5;1;2]. This was done with the aim of selecting them in the most generalizable way and to avoid consuming important computational resources to tune them automatically. (iii) Non‐maximal suppression (NMS), which quantifies the degree of overlap tolerated between two distinct objects, was set to 0.1, making it possible to have a slight overlap between detected objects. (iv) Maximum number of objects per image: During the training process, 512 objects among the ones with the best *objectness* were used to train the segmentation component.

The training of the model was run on a GeForce RTX 2070 (NVIDIA, Santa Clara, California, USA) using stochastic gradient descent with the following parameters: a batch size of 2, the total number of epochs set to 40, and a learning rate of 0.001. The evaluation of the most appropriate epoch number was performed on the validation set during the training phase. We use a *warmup strategy*, where the learning rate increases linearly from 0.0005 to 0.001 during the first epoch. To improve the invariance and robustness of the model, we applied a large set of data augmentation techniques including random horizontal and vertical flips, random rotations, and random variations in color contrast, saturation, brightness, and hue color values. To prevent overfitting and underfitting by the model, we made extensive use of batch normalization (Ioffe and Szegedy, 2015), which is currently the most effective and popular regularization technique in deep learning.

### Description of experiments

Based on the predicted and expected object instances of the test set, we computed the following evaluation measurements:

- *Average precision at a fixed intersection over union value*: This is a common metric used to evaluate instance segmentation tasks, in particular in the context of the popular MS COCO challenge (http://cocodataset.org/#detection‐eval [accessed 2 June 2020]) (Lin et al., 2014) which is the most authoritative benchmark in the field of object recognition and detection. The first step consists of determining, for each object of the ground truth (i.e., the desired output prediction of an algorithm on a specific input), all the candidate detections that have sufficient overlap with it. This is done by computing the union and intersection of the object’s masks and keeping only the predicted masks that have an *intersection over union* (IoU) value above a fixed threshold (in our case, IoU > 50%). Then, for a given class (i.e., a given species in our case), all the remaining matches are sorted by decreasing confidence in the prediction (i.e., by the maximum probability of the softmax output of the classifier). Finally, the *average precision* (AP) is computed from that sorted list according to:

$$AP\;=\;\frac{\sum_{k=1}^{n}P\left(k\right)\delta \left(\hat{y}k=yk\right)}{N_{\mathrm{gt}}}$$

where *N*_gt_ is the number of object instances in the ground truth, δ(.) is an indicator function equaling 1 if the predicted label of the detected object is equal to the ground truth label, and *P*(*k*) is the *precision* measured over the top‐*k* results (i.e., the number of correct matches in the top‐*k* first detections divided by *k*).

- *Size‐wise AP*: The AP regarding the size of objects is defined as follows: small—an area between 0 and 3.5 cm^2^; medium—an area between 3.5 and 9.5 cm^2^; and large—an area higher than 9.5 cm^2^.
- *Detection and confusion probability matrix*: This is a matrix that gives the probability of detecting a specimen of a particular species and the probability of misclassifying it as another species. It was computed based on the best match of each specimen regarding the prediction score (i.e., softmax output).
- *Error of the barycenter position*: As discussed earlier, the barycenter of the plant may be considered as a good positioning point for precise weed removal processes such as electrification. We therefore computed the average spatial distance between the barycenter of the masks in the ground truth and the predicted masks, and considered the predicted masks that had the best IoU with the ground truth to contain a correct label. Moreover, to fairly evaluate the benefit of considering masks rather than bounding boxes, we also computed this error by using the center of the bounding box of the predicted mask rather than the barycenter. Distances were first computed in pixels and then converted to millimeters using a calibration of the image size with regard to the real respective field.

## RESULTS

### Heterogeneity of performance across species

**Figure 5:**
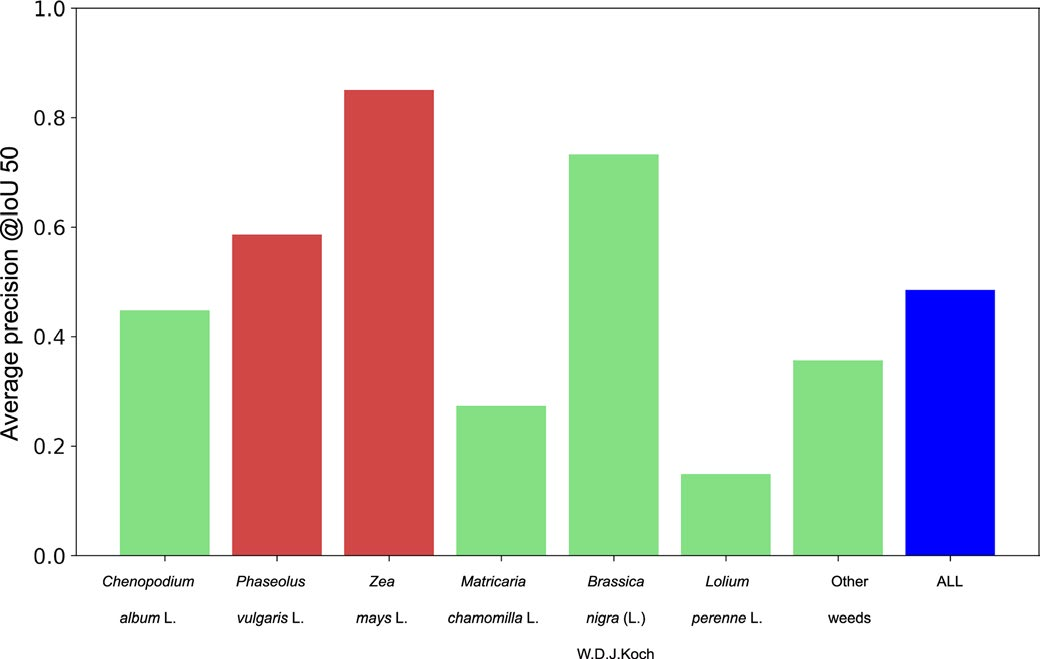
Average precision per species for an intersection over union (IOU) value of 50%. Crops are in red, weeds in green, and the mean average precision over all classes in blue.

Figure 5 displays the AP of the model predictions for the two targeted crops, the four targeted weeds, and the additional weeds category referred as *other weeds*. A high variability of performance can be observed across the different categories of plants. The best AP was obtained for *Zea mays* (AP = 0.85), demonstrating the very good detection, segmentation, and identification of most specimens of that crop. Among the remaining species, *Phaseolus vulgaris* and *Brassica nigra* were also well detected, with APs equal to 0.59 and 0.73, respectively. The other three targeted classes were more difficult to detect; the resulting AP was 0.45 for *Chenopodium album*, 0.36 for other weeds, and 0.27 for *Matricaria chamomilla*. The lowest score was obtained for *Lolium perenne*, which had an AP of 0.15. The mean AP across all categories was 0.49.

### Impact of plant size

**Figure 6:**
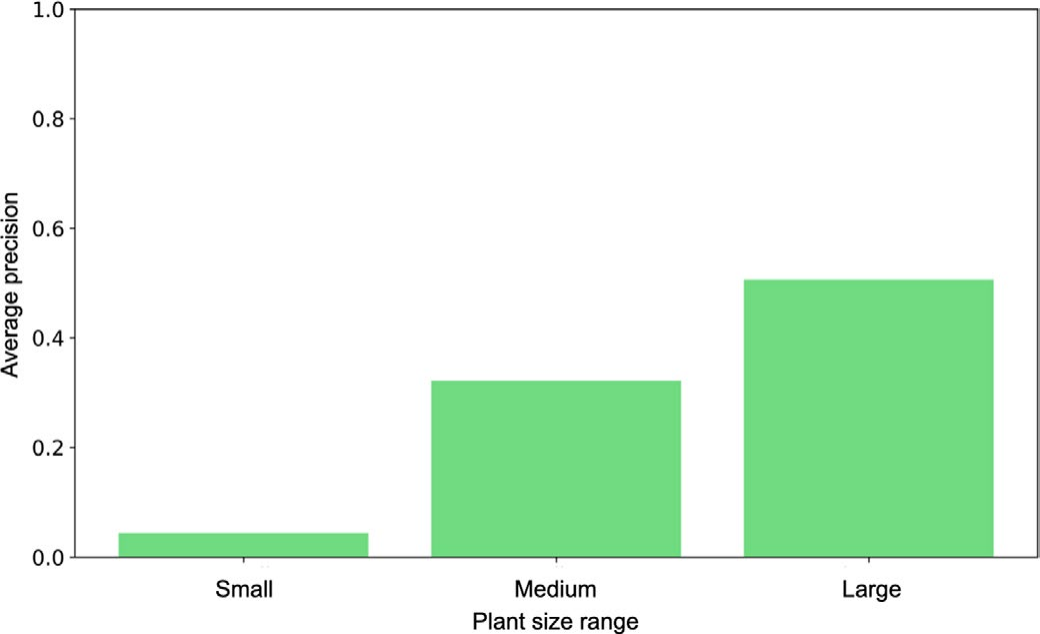
Average precision by plant size: Small plants—area between 0 and 3.5 cm^2^; medium plants—area between 3.5 and 9.5 cm^2^; large plants—area higher than 9.5 cm^2^.

**Figure 7:**
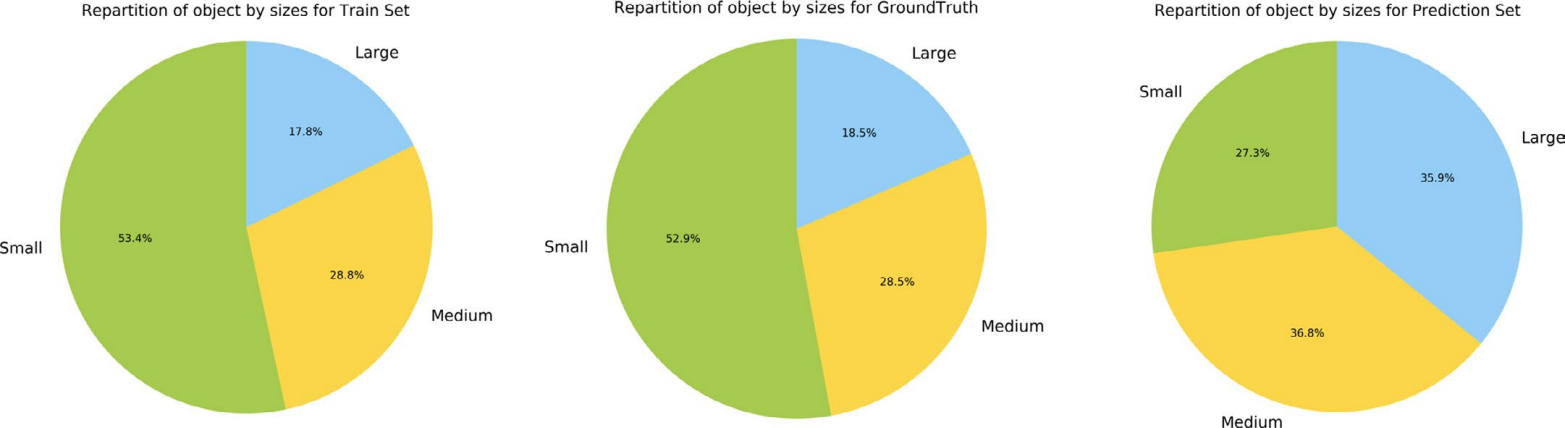
Percentage of objects of each size in the training set, the ground truth, and the test set. For the test set, we considered the 101,018 instances with the best prediction score (in order to have the same number of instances as in the test set).

To better understand the variability of performance, Fig. 6 displays the AP values broken down by plant size category rather than by plant type. As we can see, plant size had a significant impact on performance. Plants annotated with a mask larger than 9.5 cm^2^ were detected with an AP of 0.51, while the smallest plants (less than 3.5 cm^2^) were detected with an AP of 0.22. It is important to remember here that the hyperparameters of our model were chosen in such a way as to cover all sizes of objects present. The higher probability of misdetection for the small objects is thus likely to be due to a bias rather than a problem of resolution. This was confirmed by the statistics presented in Fig. 7, which show the percentages of objects of each size in the training set, the ground truth, and the predictions. As we can see, at equivalent numbers of plant instances, the proportion of small predicted objects is much lower than in the ground truth (whereas the training and test sets have similar statistics). In future work, we will investigate this problem more deeply to determine the provenance of the bias (e.g., a bias in the anchor sampling step or in the objectness measure) leading to detection/segmentation issues such as a single large object being detected instead of two small ones.

### Applicability of the method

**Figure 8:**
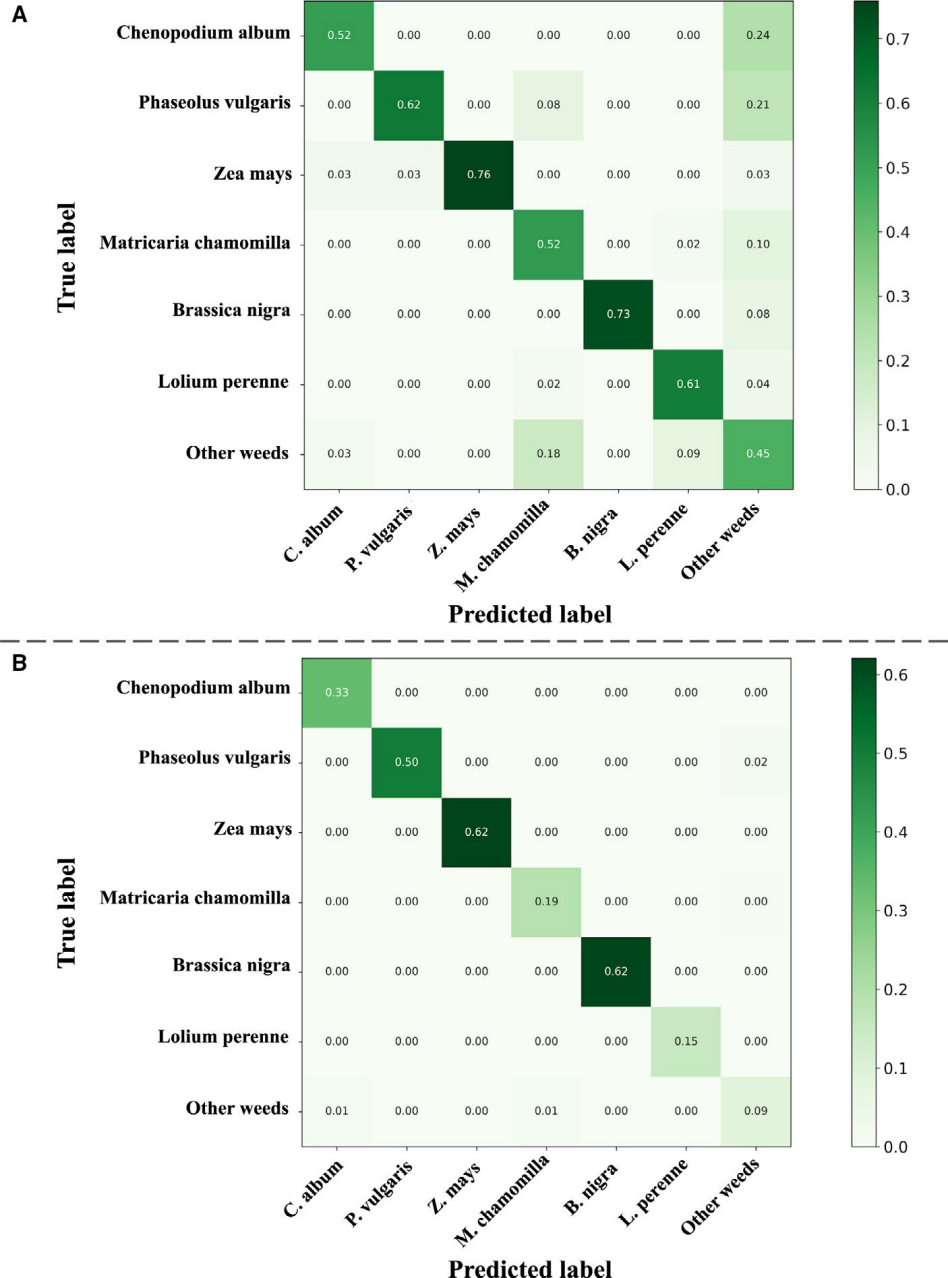
Detection and confusion probability matrix for two different operating points. (A) Top matrix: threshold = 0.1. (B) Bottom matrix: threshold = 0.6. Each row gives the probability of classifying a plant of a given species into the set of all possible species.

To measure the applicability of this method for weed removal, Fig. 8 provides the matrix of the probabilities of detection and misclassification for two different operating points. (Operating points can be seen as specific points within the operation characteristic of the robot.) The first operating point corresponds to the case in which the system returns, on average, as many detections per image as in the training set. This point is based on a probability threshold of 0.1. The second operating point corresponds to a stricter thresholding promoting precision rather than recall. This point is based on a probability threshold of 0.6. The most critical values in these matrices are the diagonal values of weed species and the intersections of crop rows and weed columns. The former give the probability of detecting specimens of a particular weed species and, consequently, a max‐bound estimate of the probability of eliminating them. The intersections of crop rows and weed columns give the probability of misclassifying a particular crop as a particular weed and, consequently, a max‐bound estimate of the risk of removing a crop during the weeding operation.

**Figure 9:**
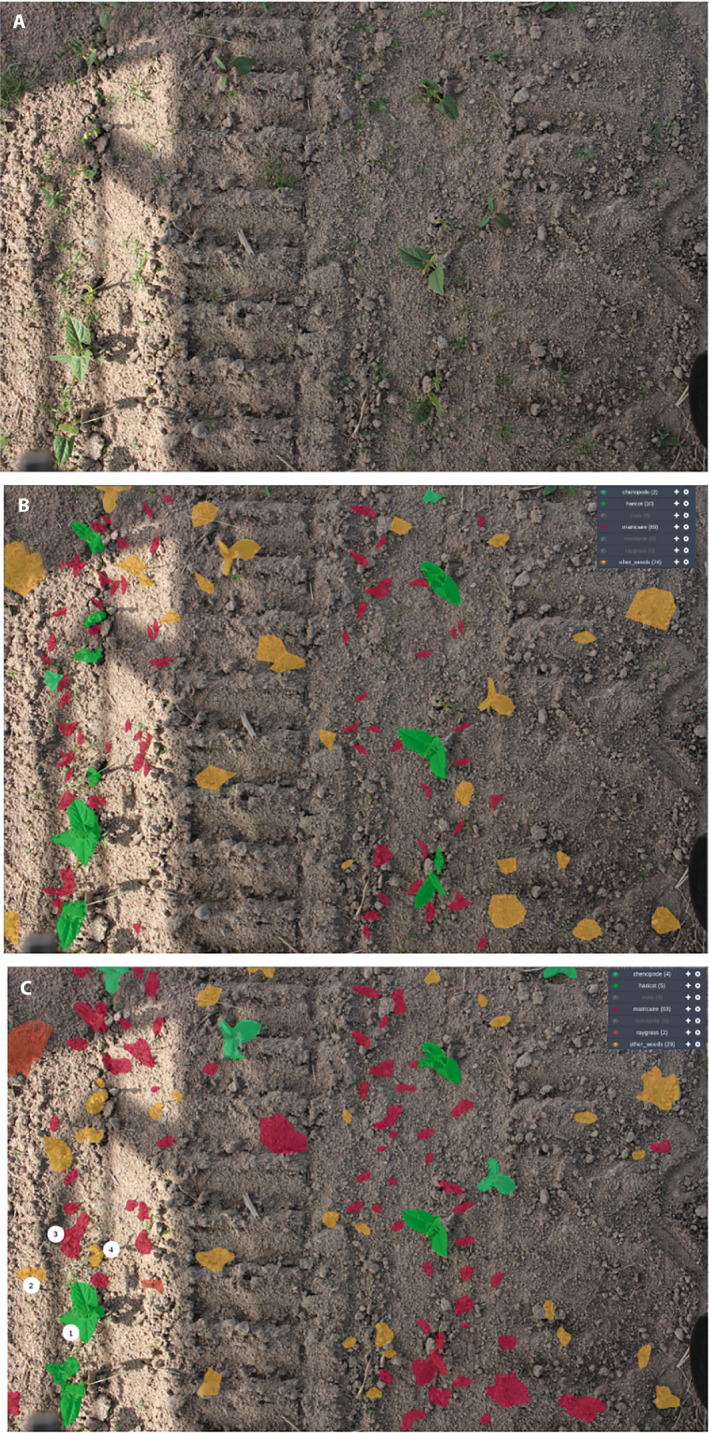
Illustration of the detection results. (A) Input image acquired by the robot. (B) Ground truth (specimens annotated manually). (C) Automatic detection results showing four sample cases: (1) an example of a correctly detected bean (*Phaseolus vulgaris*), (2) an example of a misclassified bean (*Phaseolus vulgaris*), (3) an example of a correctly detected weed, (4) an example of two weeds detected as a single weed.

The matrix in Fig. 8A (corresponding to the first operating point) shows that 45% to 73% of the weeds may be removed if one tolerates that 6% to 29% of the crops may also be erroneously eliminated. Such a loss rate may initially seem too high, but this conclusion can be moderated if we look more closely at the crop specimens that have been misclassified. Indeed, most of them usually correspond to outliers such as deteriorated, unhealthy, or degenerated individuals (see Fig. 9 for a few examples), the removal of which may not necessarily lead to a decrease in yield. The bottom matrix shows that 9% to 62% of the weeds may be removed if we use a more secure operating point at which only 0% to 2% of crops may be eliminated by error.

### Spatial accuracy of detection

We computed that the average error is 6.1 mm using the bounding box center and 2.2 mm using the predicted mask barycenter. This shows that using instance segmentation instead of a classical bounding box detection approach enables the much more accurate prediction of the true barycenter position (i.e., the “mean centroid”). In the context of weed electrification, an error of 2 mm instead of 6 mm could lead to a much more precise positioning of the electrode and, consequently, a much more efficient elimination of the weed.

## DISCUSSION

The purpose of this work was to develop and test an instance segmentation method for the fine detection of weeds and crops in precision agriculture. In particular, our aim was to evaluate the possibility of implementing electrification‐type weeding, which requires high detection and identification accuracy. The main outcome of our study is that deep learning technologies such as mask R‐CNN could be beneficial in automatic weed removal but there is still a margin for improvement regarding detection performance. To further answer the question of the current benefit of this technology, it will be necessary to set up weed removal experiments in the field.

We showed that the smaller the weeds, the higher the probability of misdetection; however, this may not necessarily be an issue for setting up innovative weeding practices. Autonomous robots in particular could have a higher passage frequency, meaning that weeds missed in the first pass could be eliminated in subsequent passes. Moreover, a partial elimination of the weeds could be sufficient to achieve high yield returns. This highlights the necessity of measuring the performance of an end‐to‐end weed removal process, with quantitative indicators such as the produced crop biomass.

The precise segmentation and identification of weeds at the specimen level is possible thanks to recent advances in deep learning, such as instance segmentation CNNs. This opens many opportunities for adapting and optimizing the treatment of each specimen. As shown in our study, it is possible to detect the barycenter of each specimen with a precision of 2 mm, enabling the use of innovative weeding approaches such as the electrification of the plant. Given that we observed that a distance of less than 1.5 cm between the weed and the electric head triggered an electric arc, the precision of these results allows this approach to be considered as a significant tool for weed control in the future.

Increasing the diversity and quantity of training data will be a necessity. It is important to remember that the performance obtained in this research was based on a rather small training data set of 83 images acquired in a single year using a small number of devices, and with specific agricultural practices. The performance could be considerably enhanced by enriching the data set. Diversifying the acquisition conditions (e.g., different sites, dates, agricultural practices), in particular, will be necessary to improve the robustness and genericity of the approach. A collaborative approach involving a more diverse group of farmers seems to be the most promising solution. Furthermore, increasing the taxonomic coverage will be essential for wide acceptance, considering that, in Western Europe alone, several hundred weed species exist (Munoz et al., 2017). The scalability of our approach will only be possible with the substantial involvement of all the key actors in weed science, who are able to detect and recognize a large number of weed species at their early stages.

There is still significant room for improvement in detection. In addition to future progress in terms of deep learning architectures, many additional improvements could be implemented and tested. In particular, a priori knowledge such as the size of the target species could greatly improve their detection (and correct biases such as the one observed in the framework we used, which is illustrated in Fig. 7, i.e., the fact that a proportion of small plants are much more important in the training set than in our test set). The fact that crops are planted in rows could also be used as a priori knowledge and greatly improve the detection and preservation of the crops. Possible improvements could also be achieved in a variety of ways, including setting up stronger acquisition conditions (e.g., artificial lighting, use of a vision chamber [i.e., a work space providing controlled conditions for image acquisition]), obtaining higher‐resolution images, or using a waterproof camera closer to the ground.

More generally, it could be beneficial to regulate the weeding action of the robot based on criteria other than the detection of weeds alone. A better understanding of the interactions between crops and weeds could, for instance, allow the determination of which species should be removed at which growth stages, and what intensity of electrification should be used based on parameters such as weed size or time of year. The growth stage and health status of the crop could also be automatically determined in order to adapt the treatment. Our work could then contribute to and benefit from the wide range of research activities conducted on the evaluation and development of machine learning techniques in plant phenotyping (Singh et al., 2018; Ruiz‐Munoz et al., 2020), crop protection (Van Evert et al., 2017), plant biology (Goëau et al., 2020; Mahood et al., 2020), and taxonomic studies (Little et al., 2020; Saryan et al., 2020).

## Supporting information


**APPENDIX S1.** Location and overview of the experimental site. (A) Location of the Montoldre experimental site in central France. (B) Aerial view of the IRSTEA research center at Montoldre, with the 4‐ha experimental field highlighted in red. (C) Photograph of the experimental field with the robot in action. (D) Two rows of young maize plants (*Zea mays*), seeded with *Chenopodium album* in the same rows.

**APPENDIX S2.** Sketch of the modified ecoRobotix agricultural robot used in this study, showing the position of the cameras for weed detection and the location of the electric head.

## Data Availability

Data used in this study are accessible on Zenodo (Champ et al., 2020), a free and open platform for preserving and sharing research output.

## Appendix 1 Cultivation protocol used in this study.

- 20 February 2019: First ground treatment with an automated spade.
- 4 April 2019: Second ground treatment with an automated spade.
- 26–29 April 2019: Heat treatment at 1450°C with a cutilight (PIRO‐PTRF model, marketed by MME Environnement, Veuilly‐la‐Poterie, France) traveling at 1.5 km/h for the first treatment and 600 m/h for the second treatment.
- 30 April to 13 May 2019: Sowing of *Zea mays* L. and *Phaseolus vulgaris* L. in specific rows (200 m long × 1 m wide). Weeds were sown inter‐ and intra‐row at a density of 54 seeds per linear meter (for higher‐density zones) and 27 seeds per linear meter (for lower‐density zones).
- Zea mays: two rows per plot, 45,000 plants per hectare, inter‐seed spacing of 30 cm, and inter‐row spacing of 75 cm.
- *Phaseolus vulgaris*: three rows per plot, 190,000 plants per hectare, inter‐seed spacing of 14 cm, and inter‐row spacing of 37.5 cm.
- Installation of a plastic film to protect seedlings against birds and insects (Biofilm [Polystar Plastics Ltd., Northam, UK], 1.5 m wide × 250 m long).
- 22 May 2019: Visual training data acquisition.
- 23 May 2019: Visual test data acquisition.
