# Supplementary material for: Instance segmentation for the fine detection of crop and weed plants by precision agricultural robots
Source: Appl Plant Sci. 2020 Jul 28;8(7):e11373. doi: 10.1002/aps3.11373 (PMC7394709; doi:10.1002/aps3.11373)

DOI 10.1002/aps3.11373

APPENDIX S1. Location and overview of the experimental site. (A) Location of the Montoldre experimental site in central France. (B) Aerial view of the IRSTEA research center at Montoldre, with the 4-ha experimental field highlighted in red. (C) Photograph of the experimental field with the robot in action. (D) Two rows of young maize plants (*Zea mays*), seeded with *Chenopodium album* in the same rows.

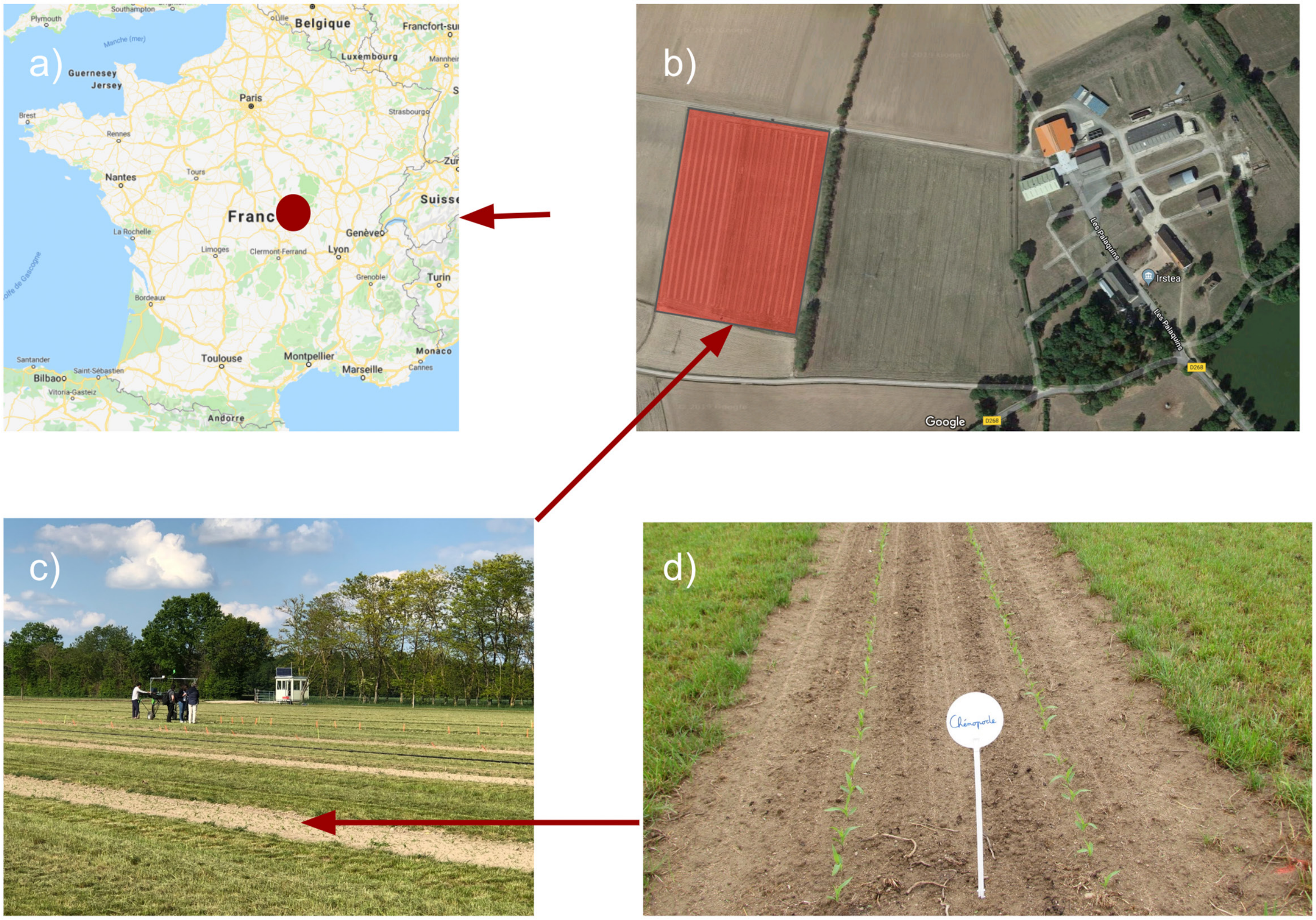

Supplement: Supplementary file 1 — APPENDIX S1. Location and overview of the experimental site. (A) Location of the Montoldre experimental site in central France. (B) Aerial view of the IRSTEA research center at Montoldre, with the 4‐ha experimental field highlighted in red. (C) Photograph of the experimental field with the robot in action. (D) Two rows of young maize plants (Zea mays), seeded with Chenopodium album in the same rows. [file APS3-8-e11373-s001.pdf]
