# Supplementary material for: Instance segmentation for the fine detection of crop and weed plants by precision agricultural robots
Source: Appl Plant Sci. 2020 Jul 28;8(7):e11373. doi: 10.1002/aps3.11373 (PMC7394709; doi:10.1002/aps3.11373)

DOI 10.1002/aps3.11373

APPENDIX S2. Sketch of the modified ecoRobotix agricultural robot used in this study, showing the position of the cameras for weed detection and the location of the electric head.

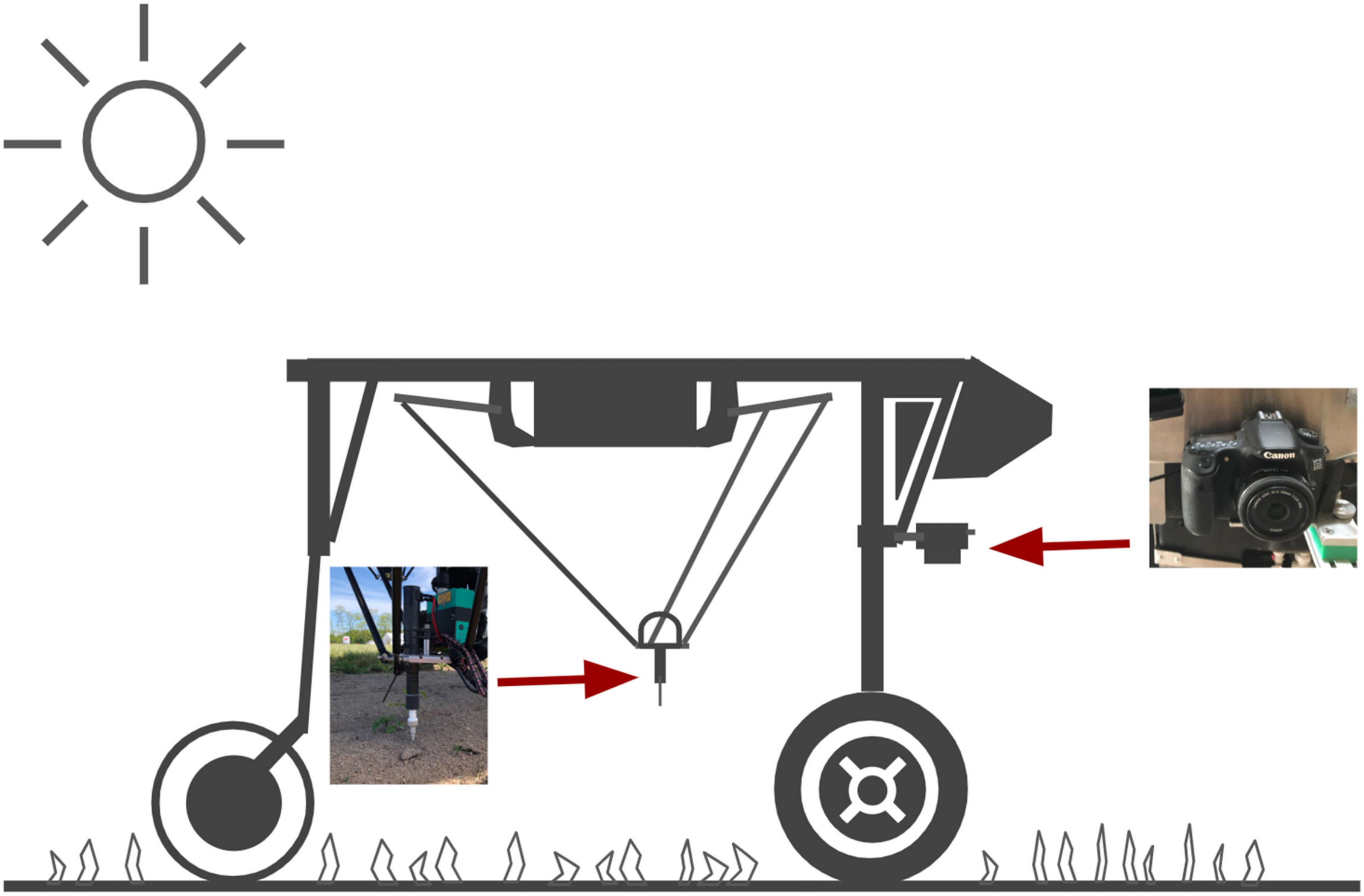

Supplement: Supplementary file 2 — APPENDIX S2. Sketch of the modified ecoRobotix agricultural robot used in this study, showing the position of the cameras for weed detection and the location of the electric head. [file APS3-8-e11373-s002.pdf]
